# Use of a Novel Anti-Infective Noble Metal Alloy-Coated Titanium Orthopedic Nail in Patients with Open Fractures: A Case Series from Malaysia

**DOI:** 10.3390/antibiotics11121763

**Published:** 2022-12-07

**Authors:** Thevarajan Karupiah, Aik Peng Yong, Ze Wee Ong, Heng Keat Tan, Wei Chern Tang, Hishamuddin Bin Salam

**Affiliations:** Orthopaedic Department, Hospital Sultanah Aminah, Jalan, Persiaran Abu Bakar Sultan, Johor Bahru 80100, Johor, Malaysia

**Keywords:** fracture-related infection, open fracture, noble metals, anti-infective, titanium

## Abstract

Fracture-related infection is a serious complication in orthopedic surgery with severe consequences for the patient. We evaluated whether a novel noble metal nail-coating technology can prevent bacterial adhesion and biofilm formation without interfering with bony union. In this retrospective, single-center case series, we described the incidence of fracture-related infections and bony union achievement in patients who had Gustilo type IIIa or IIIb femoral or tibial fractures treated with noble metal alloy-coated titanium nails. Patients were treated between January 2017 and January 2019 at the Sultanah Aminah Hospital, Johor Bahru, Malaysia. Information on fracture-related infections and bone healing assessments was collected from patient records. Additionally, three independent experts retrospectively reviewed patient X-ray images from follow-up visits to further evaluate bony union achievement. Thirty-five patients were included. Infection developed in 3/35 (8.6%) patients; all cases were resolved by antibiotic therapy. Radiographs were available for 32 patients; these confirmed the presence of bone healing in 30/32 (93.8%) patients. However, according to patient records, bony union was achieved in all patients. No safety issues were recorded. This case series suggests that a noble metal alloy-coated titanium nail can prevent infection and facilitate bony union achievement in patients undergoing surgery for severe open fractures.

## 1. Introduction

High-energy trauma, such as road traffic accidents or falls from height, often causes open fractures to the long bones, which can result in an increased risk of fracture-related infection [1]. Open fractures have an increased risk of infections compared with closed fractures; for instance, in low- and middle-income countries, the risk of infection is 3.2 times higher in patients with open fractures compared with closed fractures [2]. A common method to treat long-bone fractures is intramedullary (IM) nailing, which minimizes further soft tissue damage and facilitates bone union and healing [1]. However, a major complication in surgery is postoperative fracture-related infection, which may lead to nail failure, revision surgeries, nail removal and, in severe cases, amputation or death [3]. There is limited information available on fracture-related infections in resource-limited settings, and a 2022 review noted that 78.5−100% of authors on fracture-related infection consensus guidelines were from high-income countries [4]. Nevertheless, a large, international prospective study using an international online database called the “Surgical Implant Generation Network” (SIGN) found that 11.9% of patients with IM nail-treated open tibial fractures treated in low- and middle-income countries developed fracture-related infections [5]. In particular, infection rates were higher for Gustilo type II (12.6%), type IIIa (12.5%), type IIIb (29.1%) and type IIIc (16.7%) fractures compared with type I (5.1%) fractures [5]. The study included 1061 open tibial fracture cases between March 2000 and February 2013 [5]. Other than noting that the cases were from low- or middle-income countries, the authors did not specify which countries the cases originated from [5]. Additionally, a single-center randomized clinical trial in Tanzania found that 13.5% (15/111) of IM nail-treated patients with open tibial fractures underwent revision surgery for deep infections [6]. 

Fracture-related infections are caused by bacterial adhesion and subsequent biofilm formation on the nail surface [1,7], both of which can be prevented by adding an anti-infective nail coating [8]. Silver coating, due to its antimicrobial properties, has been used extensively for infection prevention [8,9]. A previously developed non-releasing noble metal alloy (gold, silver and palladium) medical device coating (OrthoSyn™, Vigilenz Medical Devices, Malaysia owned by Bactiguard AB, Tullinge, Sweden) has demonstrated favorable outcomes when applied on urinary catheters [10,11,12,13,14,15,16,17], endotracheal tubes [18,19] and central venous catheters [20]. The primary mechanism of action of the coating is thought to be galvanic in nature [21]. In vivo evaluation of soft tissue peri-implant reactions to noble metal coatings exhibited superiority of noble metal alloy coatings containing gold, silver and palladium compared with other coatings, and biocompatibility and anti-inflammatory properties compared with an uncoated control [22]. The noble metal alloy coating, when applied to a titanium nail, has also demonstrated nearly complete inhibition of *Staphylococcus aureus* adhesion in vitro [21]. 

Antibiotic-coated nails have also been associated with infection prevention and relatively high rates of bony union achievement in patients with tibial open fractures (34/38 patients; mean follow-up period (range): 24.1 (18–43) months) [23]; however, due to the worsening antibiotic resistance crisis, infection prevention strategies that do not involve antibiotics are becoming more important to lessen the high clinical and economic burden associated with orthopedic infections [24,25,26].

This case series aimed to evaluate clinical outcomes of a noble metal alloy-coated titanium nail in patients who underwent surgery for femur or tibia Gustilo type IIIa and IIIb open fractures. The results of this study suggest that a noble metal alloy coating on titanium nails can prevent infections and do not interfere with bony union formation in patients with severe orthopedic open fractures. 

## 2. Results

A total of 36 patients were identified for this case series. One patient defaulted and was subsequently excluded from the study due to a lack of available data. Thirty-five (27 male) consecutive patients (mean age of 33 years [range: 14−75]) with isolated tibial (74.3%, *n* = 26) or femoral (25.7%, *n* = 9) open fractures were included in this case series. All patients were victims of road traffic accidents. Most patients underwent primary wound closures (82.9%, 29/35) and the remaining patients received vacuum-assisted closures (17.1%, 6/35). Of the patients who underwent vacuum-assisted closure, two underwent one vacuum dressing cycle, two underwent three cycles, and two received daily dressing (number of cycles not specified). Radiographs were available for the independent review for 91.4% (32/35) of patients. Fracture classifications and other patient characteristics are outlined in Table 1. 

### 2.1. Infections

Infection developed in 8.6% (3/35) of patients and was resolved by antibiotic therapy in all cases. 

The first patient, an 18-year-old male, had an Arbeitsgemeinschaft für Osteosynthesefragen (AO) type 42 B2 Gustilo IIIa open fracture. The infection, caused by *S. aureus*, was diagnosed 4 months after the surgery. The duration of antibiotic therapy required for resolution was not recorded.

The second patient was a 61-year-old male with a history of diabetes mellitus, whose Gustilo type IIIa open fracture was classified by independent experts as AO 42 B3. The infection was caused by *Escherichia coli* and detected 2 months after surgery. One year of antibiotic therapy was required before sinus closure. 

The third patient, a 32-year-old male with an AO 42 C2 Gustilo type IIIb open fracture, developed a polymicrobial infection caused by both *S. aureus* and *Pseudomonas aeruginosa*. The infection was diagnosed 2 weeks after surgery and resolved following 1 month of antibiotic therapy. The patient underwent dynamization and revision surgery; wound debridement, washout and nail removal were performed. After 3 months, the coated nail was re-interlocked. Further details on the patients who developed a fracture-related infection can be found in Table 2.

### 2.2. Bony Union Assessment by the Treating Orthopedic Surgeons

Bony union, as assessed by the treating orthopedic surgeons, was achieved in all patients. Four patients (11.4%) underwent dynamization (at 6 weeks, 2 months, 5.5 months and 6 months post-operatively), and only one patient (2.9%) required revision surgery 13 months after the first surgery before achieving bony union. The mean time to union was 6.4 months (range: 3−16 months).

No adverse reactions related to the noble metal alloy coating were reported.

### 2.3. Bony Union Assessment by the Independent Expert Panel

Out of the 35 patients treated, only 32 patients (91.4%) had radiographs available for retrospective review by the three-expert panel. After review of the available radiographs, the experts unanimously agreed that 18.8% (6/32) of patients had achieved bony union at 6 months post-operatively, which increased to 93.8% (30/32) by each patient’s last recorded follow-up. As 43.8% (14/32) of patients were still on follow-up at the time of data collection, the mean time to last follow-up could not be calculated; however, the shortest follow-up duration was 40 days. The patient with the longest follow-up period was still under evaluation at the time of data collection; this patient had been on follow-up since 14 March 2017.

**Figure 1:**
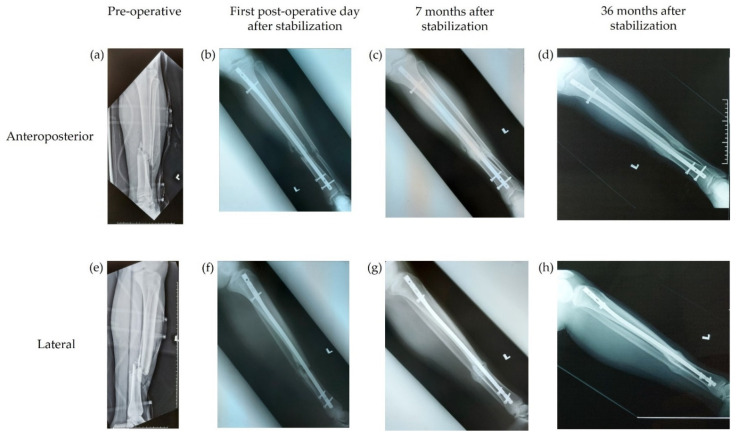
Example of timely bony union, as assessed by the independent experts. Radiographs from a 64-year-old male patient with a Gustilo type IIIa open tibial fracture. (**a**–**d**) Anteroposterior radiographs. (**e**–**h**) Lateral radiographs. (**a**,**e**) Radiographs taken pre-operatively; (**b**,**f**) radiographs taken on the first post-operative day after stabilization of the fracture; (**c**,**g**) radiographs taken 7 months after stabilization and demonstrate bony union; and (**d**,**h**) radiographs taken 36 months after stabilization.

**Figure 2:**
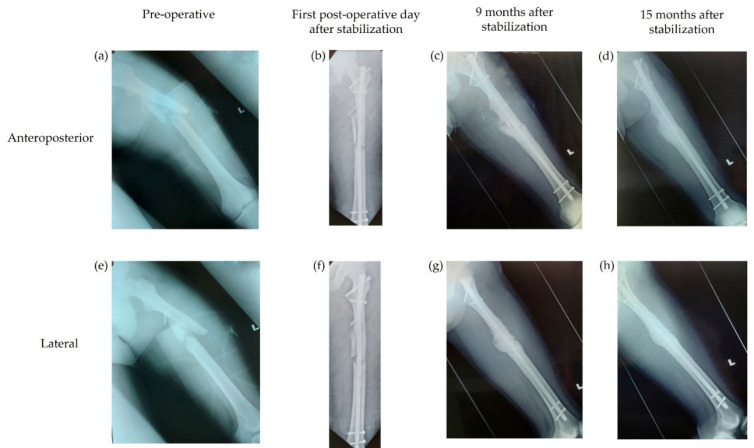
Example of delayed bony union, as assessed by the independent experts. Radiographs from an 18-year-old male patient with a Gustilo type IIIa open femoral fracture. (**a**–**d**) Anteroposterior radiographs. (**e**–**h**) Lateral radiographs. (**a**,**e**) Radiographs taken pre-operatively; (**b**,**f**) radiographs taken on the first post-operative day after stabilization of the fracture; (**c**,**g**) radiographs taken 9 months after stabilization; and (**d**,**h**) radiographs taken 15 months after stabilization that demonstrate bony union.

One of the two patients who did not achieve bony union by their last hospital visit, according to the expert evaluation, was still under review at the time of data collection. This 29-year-old male patient had an AO 32 B2 Gustilo type IIIa femoral open fracture. The second patient was a 22-year-old male with an AO 42 B2 Gustilo type IIIb tibial open fracture. Figure 1 and Figure 2 contain examples of both timely and delayed bony union, respectively, as assessed by the independent experts. 

## 3. Discussion

This retrospective case series is the first study to evaluate the anti-infective properties of a noble metal alloy coating on orthopedic nails in patients who underwent surgery for tibial or femoral Gustilo type IIIa or type IIIb open fractures. Infection occurred in a minority of cases, and a high rate of bony union achievement was recorded. No adverse reactions or safety concerns related to the noble metal alloy coating were registered. In a recent study in low- and middle-income countries, the infection rates for type IIIa and IIIb open fractures ranged between 12.5−29.1% [5]. Although not directly comparable, the present case series suggests a lower infection rate. 

Post-operative infections are associated with a high clinical and economic burden, and a reduced quality of life for patients [25,26]; thus, strategies to reduce infection rates are required. In particular, low- and middle-income countries generally have limited hospital resources, such as reduced availabilities of diagnostic tests and orthopedic specialists [27], and may significantly benefit from enhanced infection prevention strategies. Anti-infective noble metal alloy nail coatings may present an affordable and sustainable strategy for lowering the infection burden associated with orthopedic nails.

Several clinical studies have demonstrated that the incidence of post-operative infections can be significantly lowered by using devices with a silver-releasing coating, instead of uncoated devices [28,29]. These studies reported 2 to 4 times lower infection rates in patients receiving a silver-coated megaprosthesis compared with those receiving an uncoated megaprosthesis. The current case series focused on patients who received non-releasing noble metal alloy-coated orthopedic nails, therefore, comparisons with silver-coated endoprosthetic replacements is difficult. Nevertheless, the low incidence of infections in this report suggests that, in line with the results from previous studies on silver-coated megaprostheses [28,29], a non-releasing noble metal alloy-coated nail containing silver may reduce the risk of infections and subsequently lessen the clinical and economic burden associated with fracture-related infections.

Silver toxicity has been suggested as a potential risk associated with silver coatings [30]. In contrast to these silver-releasing coatings, the noble metal alloy coating of the present study is non-releasing, minimizing the risk of any toxicological effects [31]. Moreover, a recent study demonstrated that the same noble metal alloy coating used across all the nails in this case series is suitable for long-term use, with no detectable release of metal into urine [16]. 

This study was limited by the lack of a control group and the small number of patients included, which prevented the use of powered statistical analyses. Additionally, the case series did not report on changes in health-related quality of life or functional scores throughout the healing process. These are important patient-related considerations when assessing fracture healing. The retrospective nature of the expert evaluation also limited bony union assessments; expert evaluation could not be conducted for all 35 patients, as some patients did not have radiographs available for review. Furthermore, the slight discrepancies in bony union assessments between treating orthopedic surgeons and the independent experts may be partly attributed to the different bony union definitions that each group used. Unlike the treating surgeons, expert evaluation of bony union relied only upon the radiographs as they could not retrospectively test the ability of the bone to bear weight as tolerated at each state of the Perkins classification. There were also discrepancies in individual bony union evaluations amongst the experts, highlighting the difficulties in making these assessments using radiographs alone. Nevertheless, by the last follow-up, a high proportion of patients achieved bony union as assessed by both the surgeons and independent experts. 

Despite the limitations listed above, this study has multiple strengths. As the case series only included patients from one center, the influence of confounding factors, such as discrepancies in hospital procedures, was limited. Additionally, this case series provides a perspective on the influence of noble metal alloy coatings on orthopedic nail surgery outcomes in resource-limited settings, as the Sultanah Aminah Hospital is a publicly funded and resource-limited tertiary care hospital [32]. Moreover, this study provides pilot data to guide the design of future studies as, to our knowledge, this is the first study that evaluates a noble metal alloy coating on orthopedic nails.

Future studies may benefit from prospective designs, large sample sizes, and suitable controls to confirm the anti-infective properties of the noble metal alloy coating and high occurrence of bony union reported here. It may also be beneficial to evaluate the need for prophylactic antibiotics with the use of a noble metal alloy-coated nail, in addition to the long-term effects of the coating. With the increasing prevalence of antibiotic-resistant bacteria [24], infection prevention strategies that do not involve prophylactic antibiotic use, such as anti-infective nail coatings, will be required.

## 4. Materials and Methods

This single-center retrospective case series included patients who had tibial or femoral Gustilo type IIIa or type IIIb open fractures fixed using titanium orthopedic nails (titanium alloy Ti-6AL-4V) coated with the noble metal (gold, silver and palladium) alloy coating (OrthoSyn™, Vigilenz Medical Devices, Malaysia owned by Bactiguard AB) at the Sultanah Aminah Hospital, Johor Bahru, Malaysia between January 2017 and January 2019. Polytrauma cases were excluded from the study. 

All patients had undergone primary interlocking IM nailing surgery within 24 h of admission as per unit protocol and had their fractures fixed using a titanium orthopedic nail, coated with the noble metal alloy coating. The unit does not have a dedicated plastic surgery service offering microvascular soft tissue reconstruction techniques. All wound management was carried out by the local orthopedic team. Management of the soft tissue injury included primary wound closure where appropriate and vacuum-assisted devices as indicated.

The coating consists of a thin, non-continuous layer of discrete clusters that are firmly bound to the implant surface through covalent bonds. At the thickest parts, the coating is 5–10 atoms thick. The coating was applied to both the titanium nails and screws. The coating weighed ~2 µg per square centimeter for silver and 0.2 µg for gold and palladium, respectively. The total weight of the coating per nail was 238 µg. As per hospital protocol for the management of open fractures, all patients received intravenous cefuroxime 750 mg 3 times daily during their hospital stay, and when discharged were prescribed cefuroxime 250 mg (tablets) twice daily for 2 weeks.

The outcomes of this case series were the incidence of fracture-related infections, as well as the achievement of bony union. Infection diagnoses were based on the Centers for Disease Control and Prevention definition of deep surgical wound infection [33], which included at least one of the following: purulent drainage from the deep incision, presence of wound dehiscence and a positive culture (or fever >38 °C in the absence of a culture result), localized pain and tenderness. The following laboratory parameters were used to support diagnosis: erythrocyte sedimentation rate (<22 mm/h for men, <29 mm/h for women) and C-reactive protein <10 mg/L. In accordance with hospital protocols, patients were monitored for signs and symptoms of infection at follow-up visits and underwent further surgery (i.e., debridement, washout, or removal of nail) if the infection was diagnosed and could not be controlled. Bony union, defined radiologically as the presence of at least three cortices of bridging callus on the anterior, posterior, and lateral views, and clinically by the ability to bear weight as tolerated at each state of the Perkins classification of fracture healing, was also assessed by orthopedic surgeons during outpatient follow-up visits using X-ray imaging.

Three independent orthopedic trauma experts with more than 10 years’ clinical experience retrospectively reviewed patients’ X-rays to further classify the Gustilo type IIIa and type IIIb open fractures according to the AO fracture classification system [34]. Each expert produced an individual rating for each patient and final ratings were subsequently decided over a conference call. In addition, the experts radiologically assessed bony union at 6 months post-operatively and at the last recorded follow-up. 

Descriptive data were reported in frequencies for categorical variables and mean and standard deviation, or range, for continuous variables. 

## 5. Conclusions

In conclusion, the present case series suggests that a non-releasing noble metal alloy-coated titanium nail is safe and can be used for the prevention of infection and facilitation of bony union in patients undergoing surgery for femoral and tibial IIIa and IIIb open fractures. It appears that this noble metal alloy coating, with a galvanic mechanism of action, provides a valid technology to prevent fracture-related infections.

## Figures and Tables

**Table 1:** Baseline characteristics.

	Patients (*N* = 35)
Sex, *n* (%)	
Male	27 (77.1)
Female	8 (22.9)
Age, mean (SD)	33 (15)
Comorbidities, *n* (%)	
Diabetes mellitus	1 (2.9)
Smoking	1 (2.9)
Gustilo type of open fracture, *n* (%)	
Type IIIa	29 (82.9)
Type IIIb	6 (17.1)
AO fracture classification, *n* (%)	
32 A3	1 (2.9)
32 B2	3 (8.6)
32 B3	3 (8.6)
32 C2	2 (5.7)
42 A1	2 (5.7)
42 A3	4 (11.4)
42 B2	11 (31.4)
42 B3	3 (8.6)
42 C2	3 (8.6)
Unavailable	3 (8.6)
Site of fracture, *n* (%)	
Tibia	26 (74.3)
Femur	9 (25.7)

AO, Arbeitsgemeinschaft für Osteosynthesefragen; SD, standard deviation.

**Table 2:** Characteristics of patients with surgical site infection according to CDC diagnostic criteria.

Patient	AO Fracture Classification	Gustilo Type	Underlying Medical Issues	Timing of Infection from Index Surgery	Timing of Union from Index Surgery	Fever	Sinus Location	Wound Dehiscence	Warm	Erythema	Isolated Microorganism	CRP, mg/L
1	42 B2	IIIa	Nil	4 months	4 months	No	Distal screw	No	Yes	Yes	*Staphylococcus aureus*	5.50
2	42 B3	IIIa	Diabetes mellitus	2 months	8 months	No	Fracture site	No	Yes	Yes	*Escherichia coli*	6.20
3	42 C2	IIIb	Nil	2 weeks	1 year 4 months	No	No	Yes	Yes	Yes	*S. aureus &* *Pseudomonas* *aeruginosa*	8.34

AO, Arbeitsgemeinschaft für Osteosynthesefragen; CDC, Centers for Disease Control and Prevention; CRP, C-reactive protein.

## Data Availability

The data that support the findings of this study are available on request from the corresponding author. The data are not publicly available due to privacy or ethical restrictions.
